# High‐Temperature Sintering of Garnet Solid Electrolyte Li_7_La_3_Zr_2_O_12_: A Comparative Study of Induction Hot Pressing and Spark Plasma Sintering

**DOI:** 10.1002/smll.202506257

**Published:** 2025-08-28

**Authors:** Mikihisa Fukuda, Ying Li, Jing Wei, Koichi Harata, Guoqiang Luo, G. Jeffrey Snyder, Yutaka S. Sato, Hidemi Kato, Eric Jianfeng Cheng

**Affiliations:** ^1^ Department of Materials Science Graduate School of Engineering Tohoku University Sendai 980‐8579 Japan; ^2^ Advanced Institute for Materials Research (WPI‐AIMR) Tohoku University Sendai 980‐8577 Japan; ^3^ State Key Laboratory of Advanced Technology for Materials Synthesis and Processing Wuhan University of Technology Wuhan 430070 China; ^4^ Institute for Materials Research Tohoku University Sendai 980‐8577 Japan; ^5^ Department of Materials Science and Engineering Northwestern University Evanston IL 60208 USA

**Keywords:** garnet solid electrolyte, induction hot pressing, ionic conductivity, Li_7_La_3_Zr_2_O_12_ (LLZO), spark plasma sintering (SPS)

## Abstract

All‐solid‐state Li‐metal batteries are emerging as a transformative energy storage technology, driven by the demand for higher energy density and enhanced safety in electric vehicles (EVs). Among myriad solid electrolytes, garnet‐type Li_7_La_3_Zr_2_O_12_ (LLZO) has attracted significant attention due to its high room‐temperature (RT) Li‐ion conductivity (>10^−4^ S cm^−1^) and chemical stability against metallic Li anodes. Nevertheless, achieving highly dense LLZO remains challenging, as conventional sintering methods, such as pressureless sintering or hot pressing (HP), require prolonged processing times, resulting in Li loss and low densification. Spark plasma sintering (SPS) is considered a superior alternative due to its reputed ability to achieve rapid densification. In this study, induction HP and SPS are systematically compared for densifying Al‐doped cubic LLZO powder under various conditions. The findings indicate that both techniques can achieve comparable densification (≈98%) within 5 min, when pressure and temperature are meticulously controlled. Both HP‐ and SPS‐sintered samples show a high RT ionic conductivity exceeding 0.45 mS cm^−1^. These results challenge the conventional perception that SPS inherently provides superior densification. The analysis also confirms that densification is predominantly governed by pressure‐assisted heating. This work offers insights into optimizing high‐temperature sintering of dense oxide solid electrolytes for next‐generation solid‐state metal batteries.

## Graphical abstract

Simulated thermal distribution in graphite punches and dies during induction hot pressing (left) and spark plasma sintering (right).

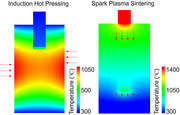

## 1 Introduction

All‐solid‐state Li‐metal batteries (ASSLMBs) are regarded as a promising next‐generation energy storage technology due to their potential for improved safety and higher energy density compared to conventional Li‐ion batteries. By replacing flammable liquid electrolytes with nonflammable solid electrolytes (SEs), the potential risk of leakage and fires is eliminated.^[^ ^1^ ^]^ They also enable the use of a Li metal anode with an ultrahigh specific capacity (≈3860 mAh g^−1^, an order of magnitude greater than graphite), which can dramatically increase cell energy density.^[^ ^2^ ^]^ Furthermore, SEs can enable bipolar cell stacking (multilayer cells without discrete separators), further boosting energy density and safety. Thus, ASSLMBs potentially offer a compelling platform for next‐generation energy storage technology, given that SEs can meet the required performance metrics.

Among various oxide SE materials, the garnet‐type Li_7_La_3_Zr_2_O_12_ (LLZO) has emerged as one of the most promising candidates.^[^ ^3^, ^4^, ^5^ ^]^ LLZO exhibits two crystal structures, tetragonal and cubic, but the cubic phase is typically stabilized at room temperature (RT) when aliovalently doped with Al (Li site) or Ta (Zr site).^[^ ^1^ ^]^ Al‐doped LLZO shows a high Li^+^ ionic conductivity on the order of 10^−4^ to 10^−3^ S cm^−1^ at RT.^[^ ^3^, ^6^ ^]^ Unlike other alternative oxide SEs such as Li_0.29_La_0.57_TiO_3_ (LLTO), LLZO is also chemically stable in contact with metallic Li anode and high‐voltage cathodes,^[^ ^2^, ^7^, ^8^ ^]^ resisting the reduction by Li or oxidation at high voltage that typically plagues other oxide (e.g., LLTO^[^ ^9^, ^10^ ^]^) and sulfide electrolytes (e.g., Li_10_GeP_2_S_12_ (LGPS)^[^ ^11^ ^]^). The combination of fast ion transport and broad electrochemical window makes LLZO a promising SE, catalyzing extensive research endeavors since its discovery in 2007.^[^ ^3^ ^]^

Despite these favorable properties, densifying LLZO powders into highly dense crystalline samples for application poses significant processing challenges. Conventional sintering methods, such as pressureless sintering, often yield incomplete densification with a relative density of less than 95%.^[^ ^12^ ^]^ Porosity in garnet‐type solid electrolytes not only reduces effective Li^+^ conductivity but also creates high‐risk sites for dendrite nucleation and propagation. Recent operando studies show that voids, whether at the Li|LLZO interface or within the bulk, can concentrate current, trap electrons, and intensify local stresses, ultimately enabling lithium filament penetration and mechanical failure of the separator.^[^ ^1^, ^13^, ^14^ ^]^ Achieving a 100% relative density is, therefore, critical for maximizing both the ionic conductivity and mechanical properties of LLZO. Ionic conductivity has been reported to positively correlate with mechanical hardness in oxide SEs.^[^ ^14^ ^]^ Besides pressureless sintering, hot‐pressing (HP) is an established approach to produce highly dense (relative density: >95%) polycrystalline LLZO, which involves sintering the powder under an applied uniaxial pressure at elevated temperatures. Since HP uses resistive heaters that radiate heat to the die externally, it often requires several hours to equilibrate the temperature.^[^ ^15^ ^]^ HP typically requires sintering temperatures in the range of 1100–1200 °C, with a holding time of several hours to achieve sufficient densification.^[^ ^16^, ^17^, ^18^ ^]^

However, prolonged high‐temperature exposure likely results in Li volatilization from the LLZO lattice​, degrading ionic conductivity.^[^ ^19^ ^]^ There is thus an inherent trade‐off in processing: insufficient sintering yields a porous, low‐conductivity ceramic, whereas excessive sintering causes Li loss and phase decomposition.

In comparison, spark plasma sintering (SPS), also known as pulsed electric current sintering (PECS) or field‐assisted sintering technology (FAST), has gained attention as a rapid densification method for LLZO.^[^ ^20^, ^21^ ^]^ In SPS, a pulsed electric current is passed through the graphite die and possibly through the cold‐pressed green body, leading to a rapid temperature rise, facilitating efficient sintering of particles.^[^ ^21^, ^22^ ^]^ Using SPS, it has been reported that dense LLZO ceramics can be fabricated in minutes rather than hours.^[^ ^23^, ^24^, ^25^ ^]^ For instance, Zhang et al. achieved 99% relative density in less than 10 min at 1150 °C via SPS.^[^ ^20^ ^]^ This drastic reduction in sintering time could minimize potential Li evaporation and the formation of Li‐deficient secondary phases. An additional benefit of SPS is that the rapid heating can inhibit excessive grain growth, presumably due to the short pressing time. However, SPS conditions change with die size, which is likely due to highly localized heat generation at high‐resistance contact points between the plunger and die.^[^ ^22^ ^]^

Induction hot‐pressing or rapid hot pressing (RHP) has a similar sintering speed as SPS to maintain a small grain size, but much more uniform die heating.^[^ ^26^ ^]^ RHP avoids the possibility of ion migration because instead of using a DC current directed at the sample, it uses a radio‐frequency (RF) induction coil that heats a few mm skin depth of the high thermal conductivity graphite die, providing a more uniform temperature at the sample. However, the densification mechanism in SPS for LLZO is not yet fully understood, and few studies have directly compared SPS with HP for SEs.^[^ ^27^ ^]^ Despite its rapid densification ability, SPS may induce temperature gradients or localized heating, compromising sintering uniformity.

In this work, we systematically compare the sintering effects of induction HP and SPS for sintering Al‐doped LLZO. We compare the densification behavior, microstructural evolution, and ionic conductivity of LLZO electrolytes produced by the two methods. By elucidating the differences in sintering outcomes between HP and SPS, this study aims to better understand the sintering mechanisms of rapid densification of LLZO using HP or SPS and provide guidance for choosing the high‐temperature sintering processes of oxide SEs for next‐generation ASSLMBs.

## 2 Experimental Section

### 2.1 High‐Temperature Sintering of Al‐Doped LLZO via HP and SPS

**Figure 1:**
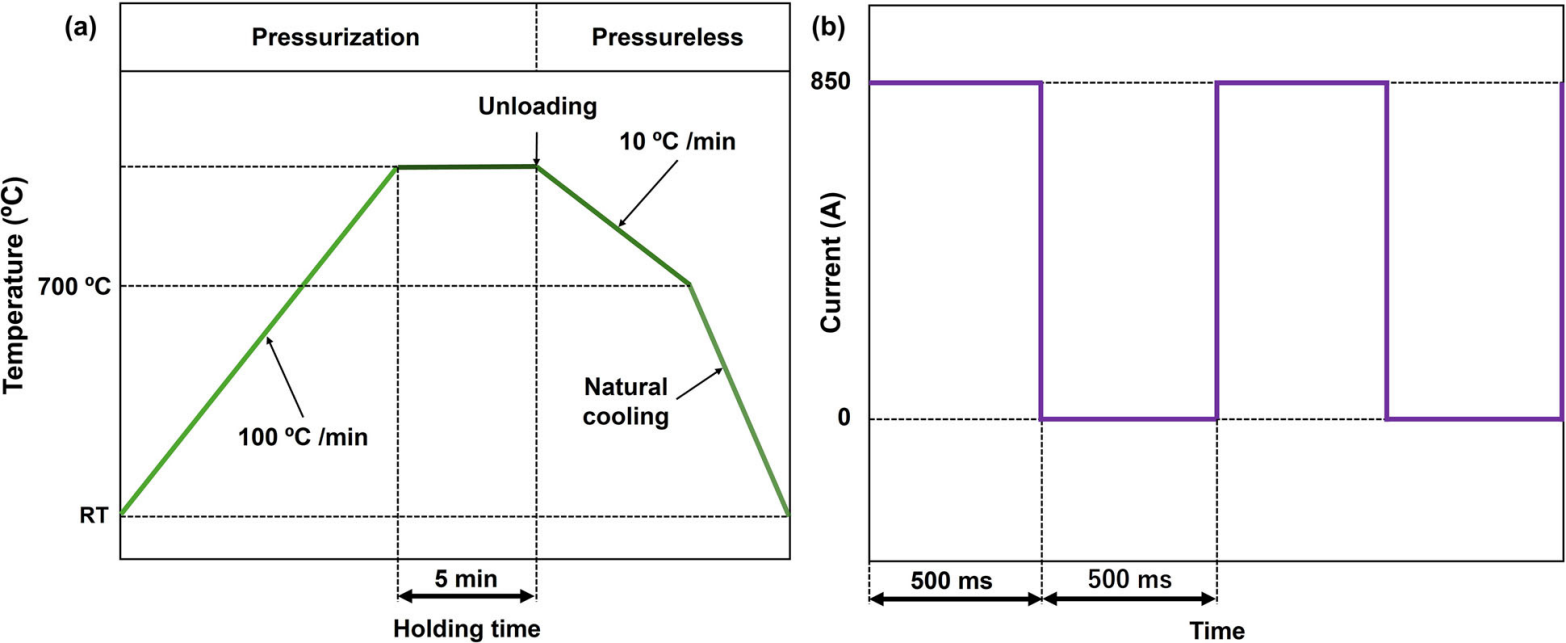
Sintering profiles. a) Sintering temperature profiles for HP and SPS, b) pulsed current profile for SPS.

Commercially available Li_6.25_Al_0.25_La_3_Zr_2_O_12_ powder (D50 = 10 µm, Daiichi Kigenso Kagaku Kogyo Co., Ltd., Japan) was used as the starting material. The powder was loaded into a graphite die and sintered via HP and SPS using a hybrid SPS system (Daiichi Kiden Co., Ltd.) under an Ar atmosphere. The hybrid SPS system is capable of performing both SPS and HP by switching the heating mode between pulsed electric current heating and high‐frequency induction heating. The LLZO powder was pre‐pressed into pellets at 10 MPa before sintering. Sintering temperatures ranged from 900 to 1150 °C in 50 °C increments, with a fixed applied pressure of 25 MPa and a fixed holding time of 5 min. In terms of heating mechanisms, SPS mainly employed pulsed electric current heating, whereas HP utilized only high‐frequency induction heating. The heating rate was set at 100 K min^−1^, followed by a controlled cooling rate at 10 K min^−1^ down to 700 °C, and natural cooling to RT. The temperature profile during sintering is shown in **Figure** **1**a, and the pulsed current profile is depicted in Figure 1b. To mitigate crack formation due to thermal shrinkage, the applied pressure was released at the onset of cooling, mitigating crack formation due to thermal contraction. The sintering temperature was monitored via an infrared (IR) thermometer directed at the graphite die surface. Carbon contamination, originating from the graphite die, was observed after sintering. To remove residual carbon, the sintered LLZO pellets underwent heat treatment at 850 °C for 4 h in air, resulting in a translucent appearance.

### 2.2 Materials Characterization

To determine the crystal structures of the sintered LLZO pellets, the samples were mechanically dry‐polished up to #2000 grit and analyzed using X‐ray diffraction (XRD) (Rigaku Ultima IV, Cu Kα radiation, λ = 1.54 Å). The XRD measurements were conducted at an operating voltage of 40 kV and 40 mA, with a 2θ scanning range of 15°–70°. For microstructural analysis, both polished and fractured specimens were examined using a scanning electron microscope (SEM) (Carl Zeiss ULTRA55) at an acceleration voltage of 15 kV. The acquired backscattered and secondary electron images were processed using ImageJ, an image analysis software developed by the National Institutes of Health (NIH), to quantify porosity and estimate the average grain size of sintered LLZO.

### 2.3 Evaluation of Relative Density

The Archimedes method was employed to determine the bulk density of the sintered samples at RT. The samples were first weighed in air after thorough drying to obtain their dry weights (A_0_). To remove trapped air within pores, the samples were immersed in deionized water and placed in a vacuum desiccator for 2 h to ensure complete saturation. The samples were then weighed (B) while fully suspended in the deionized water. After that, the samples were removed from water, lightly dried to remove surface water, and weighed again in air (A). The relative density (ρ_bulk_) is then calculated using Equation (1):

(1) $$\rho_{\mathrm{bulk}}=\frac{A_{0}}{A-B}\times \rho_{\mathrm{water}}$$

where A_0_ is the dry weight in air, A is the weight of the wet sample in air, and B is the weight of the sample in water, ρ_water_ (g cm^−3^) is the density of water at 25 °C (1.0 g cm^−3^). ρ_relative_ is determined using Equation (2):

(2) $$\rho_{\mathrm{rela}\mathrm{tive}}=\frac{\rho_{\mathrm{bulk}}}{\rho_{0}}$$

where ρ_0_ is the theoretical density of Al‐doped LLZO, 5.107 g cm^−3^.^[^ ^28^ ^]^ Although Li^+^/H^+^ exchange is expected upon exposure to moisture, it predominantly occurs near the LLZO surface. A hydrothermal aging study at 150 °C for 28 days on Al‐doped LLZO single crystals revealed Li^+^ depletion at interstitial sites along with H^+^ incorporation; however, the structural degradation remained highly localized, consistent with surface‐ or near‐surface‐limited exchange even under prolonged and aggressive conditions.^[^ ^29^ ^]^ In this study, the as‐sintered Al‐LLZO pellets were several centimeters thick and were soaked in water at room temperature for only 1.5 h. After density measurements using the Archimedes method, the pellet surfaces were thoroughly polished with sandpaper in an Ar‐filled glove box to remove any potential surface impurity layers.

### 2.4 Ionic Conductivity Measurement

The ionic conductivity of the samples at RT was determined via electrochemical impedance spectroscopy (EIS) using a BioLogic VSP‐300 system. Before measurement, a blocking Au electrode (diameter: 10 mm, thickness: ≈100 nm) was deposited onto both sides of the samples using a desktop sputter coater (Sanyu Electron SC‐701 Mk). The samples were then placed in a two‐electrode cell fixture for impedance (R) measurement at RT.

The ionic conductivity (σ, S cm^−1^) is then calculated using Equation (3):

(3) $$\sigma =\frac{L}{RA}$$

where L is the sample thickness, R is the impedance (Ω), and A is the active electrode area (cm^2^).

## 3 Results and Discussion

**Figure 2:**
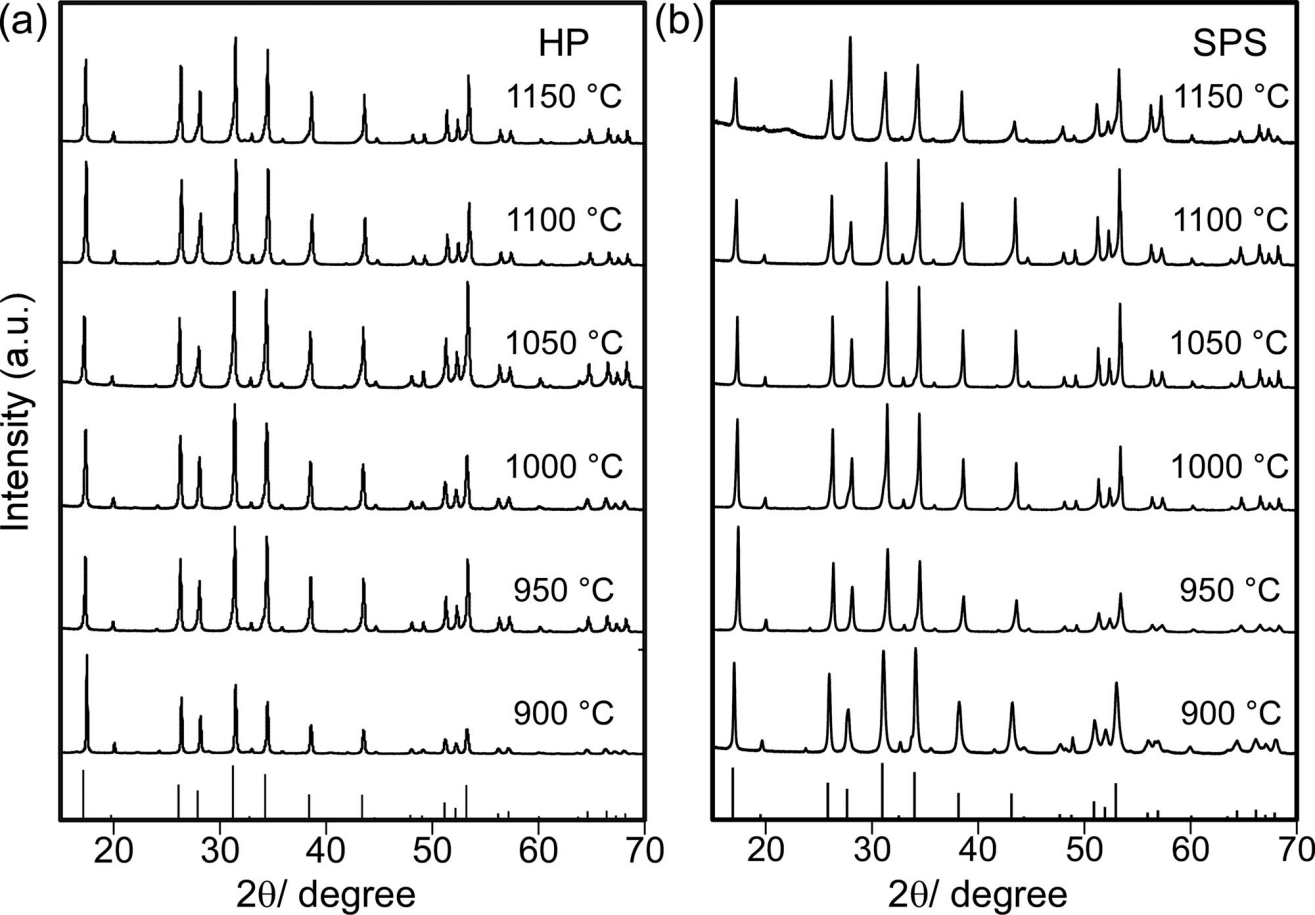
XRD patterns of Al‐doped LLZO sintered via a) HP and b) SPS in the temperature range from 900 to 1150 °C.

To investigate the influence of sintering temperatures and heating methods on the crystal structure of sintered Al‐doped LLZO, X‐ray diffraction (XRD) analysis was conducted on both the precursor powder and sintered Al‐doped LLZO pellets. The XRD patterns at temperatures ranging from 900 to 1150 °C are presented in **Figure** **2**, while the XRD pattern of the precursor powder is included as supplementary information.

The XRD pattern of the as‐received Al‐LLZO powder exhibits diffraction peaks of a typical cubic LLZO (c‐LLZO) phase, confirming its phase purity before sintering. However, a minor peak at ≈24° was observed, likely to originate from a Li_2_CO_3_ impurity phase.^[^ ^30^ ^]^ Li_2_CO_3_ is commonly used as a Li precursor during LLZO synthesis, and its formation on the LLZO surface is a well‐documented phenomenon, often resulting from exposure to atmospheric CO_2_ in the presence of moisture.^[^ ^31^, ^32^, ^33^ ^]^ When sintering at high temperatures (1150 °C for HP and 1100 °C for SPS), the minor peak disappeared. This disappearance was likely attributed to the decomposition of Li_2_CO_3_ in a reducing Ar atmosphere, where CO_2_ is actively removed from the reaction system. The decomposition process is described by the reaction (Eq. (4)):^[^ ^34^ ^]^

(4) $$Li_{2}CO_{3}\to Li_{2}O+CO_{2}↑\;\;\;\Delta G^{\circ }=\mathrm{pressure}-\mathrm{dependent}$$

Additionally, regardless of sintering temperature or method, the sintered pellets exhibited peaks exclusively corresponding to cubic LLZO, with no detectable secondary phases or decomposition products. This result demonstrates that Al‐LLZO retained its cubic phase across the entire investigated temperature range from 900 to 1150 °C, confirming the thermal stability of cubic LLZO under both HP and SPS conditions. An unexpected halo peak at ≈22° was observed in the XRD pattern of pellets sintered by SPS at 1150 °C. Based on comparison with literature data,^[^ ^35^, ^36^ ^]^ and after ruling out other potential impurity phases such as La_2_Zr_2_O_7_,^[^ ^36^ ^]^ this feature was attributed to amorphous or poorly crystalline LaAlO_3_, which can form when the Al content exceeds its solubility limit.^[^ ^35^ ^]^ Additionally, Li loss during high‐temperature sintering could possibly result in a La‐rich composition, thermodynamically favoring LaAlO_3_ formation. Localized overheating during SPS may further accelerate this reaction. Notably, LLZO typically decomposes at ≈1300 °C.^[^ ^37^ ^]^ Meanwhile, the grain‐boundary‐confined growth and the short high‐temperature dwell time likely account for its low crystallinity. The presence of LaAlO_3_ suggests that the actual local sintering temperature in SPS likely exceeded the nominal 1150 °C.

**Figure 3:**
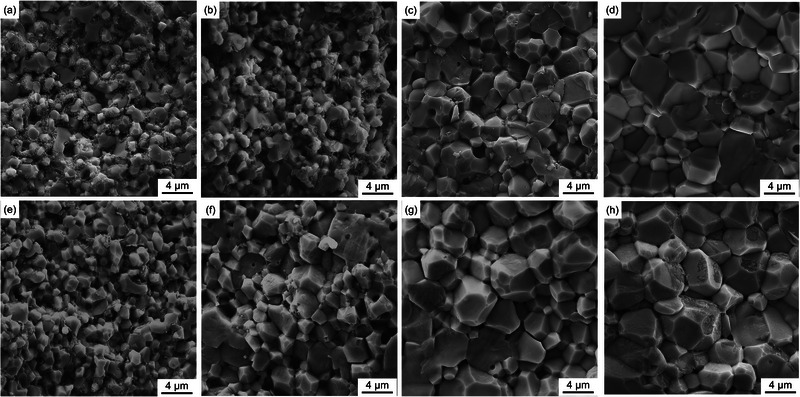
SEM images of fractured surface microstructures of Al‐doped LLZO sintered via a–d) HP (900, 1000, 1050, and 1100 °C) and e–h) SPS (900, 1000, 1050, and 1100 °C).

The microstructures of sintered Al‐LLZO pellets, particularly pore distribution and grain size, play a critical role in determining the ionic conductivity and mechanical properties. SEM microstructural images of the fractured surface, presented in **Figure** **3**, show the grain structure evolution under different sintering conditions. Quantitative grain size measurements, summarized in **Table** **1**, reveal a strong positive correlation between sintering temperature and grain size. At 900 °C, the average grain sizes of HP‐ and SPS‐sintered pellets are ≈1.6 and 1.8 µm, respectively. As the sintering temperature increases, grain growth becomes more pronounced, with the average grain sizes of HP‐ and SPS‐sintered pellets reaching 4.0 and 4.6 µm, respectively, at 1100 °C. At temperatures ≈1100 °C, both HP‐ and SPS‐sintered samples exhibit enhanced densification, with significantly reduced porosity and well‐defined grain structures. These results demonstrate that both HP and SPS can effectively densify Al‐LLZO within just 5 min at ≈1100 °C, challenging previous literature reports that SPS is significantly more efficient in densifying ceramic oxide electrolytes than HP.

**Table 1:** Average grain size of Al‐doped LLZO pellets as a function of sintering temperature and method.

Sintering temperature [°C]	900	950	1000	1050	1100	1150
Grain size in HP [µm]	1.6	1.8	2.0	2.8	4.0	5.1
Grain size in SPS [µm]	1.8	2.0	2.9	3.7	4.6	5.3

**Figure 4:**
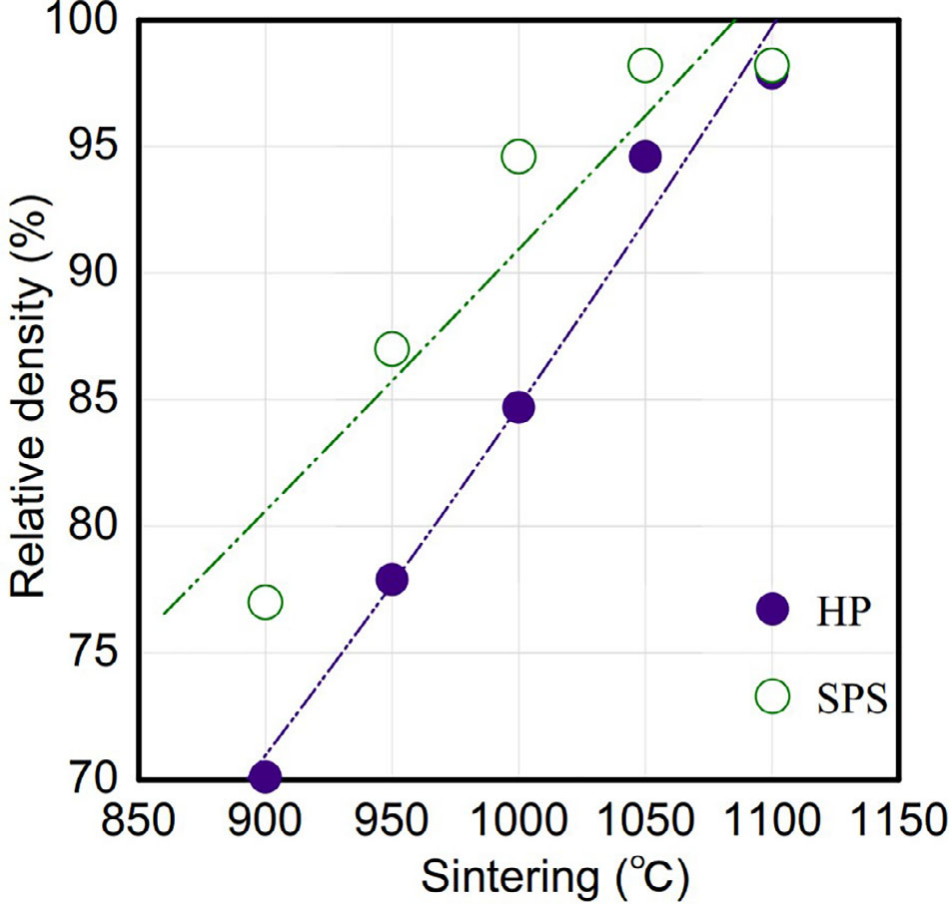
Relative density of sintered Al‐LLZO pellets as a function of sintering temperature.

The relative density of sintered Al‐LLZO pellets was evaluated using Archimedes method, with results summarized in **Table** **2** and **Figure** **4**. Both HP‐ and SPS‐sintered pellets exhibited a trend of increasing relative density with increasing sintering temperature, up to a certain threshold beyond which further temperature increases showed little effect on densification.

**Table 2:** Relative density values of Al‐LLZO pellets sintered by HP and SPS.

Sintering temperature [°C]	900	950	1000	1050	1100	1150
Relative density [%] (HP)	70.1	77.9	84.7	94.6	97.9	97.3
Relative density [%] (SPS)	77.0	87.0	94.6	98.2	98.2	98.1

For HP‐sintered samples, relative density consistently increased steadily with increasing temperatures from 900 to 1100 °C, reaching a maximum of 97.9 %. However, further densification became negligible despite further heating when the sintering temperature reached 1100 °C. A similar trend was observed in SPS‐sintered samples, where relative density increased with increasing temperatures from 900 to 1050 °C, reaching a maximum of 98.2 %, but remained nearly constant after further heating. This plateau suggests that grain growth alone, which typically occurs with increasing temperatures, does not necessarily result in higher densification.

During sintering, densification is typically driven by grain growth, pore shrinkage, and pore elimination. As confirmed by SEM micrographs in Figure 3, the grain size increases with temperature in both HP‐ and SPS‐sintered samples. However, beyond 1100 °C in HP and 1050 °C in SPS, relative density remained unchanged despite continued grain coarsening, indicating that at the final stage of sintering, grain growth becomes independent of densification. Closed pores likely become isolated within grains, and further elimination occurs through slow mechanisms such as bulk lattice diffusion rather than fast grain boundary diffusion. If grain growth proceeds without effective pore shrinkage, it can even impede densification, as larger grains tend to trap residual closed pores.^[^ ^38^ ^]^

These results show that while SPS slightly enhances densification kinetics compared to HP, both methods can achieve rapid densification of Al‐LLZO powders, challenging the assumption that SPS is significantly more effective than HP in densifying ceramic oxide electrolytes. While HP and SPS can achieve similar densification under optimal conditions, SPS generally attains a higher effective sintering temperature for the same nominal setpoint due to its rapid, localized heating via induction and Joule heating of the graphite mold. At lower sintering temperatures, where both methods yield porous microstructures, this temperature advantage becomes more pronounced, contributing to higher ionic conductivity in SPS‐prepared samples.

**Figure 5:**
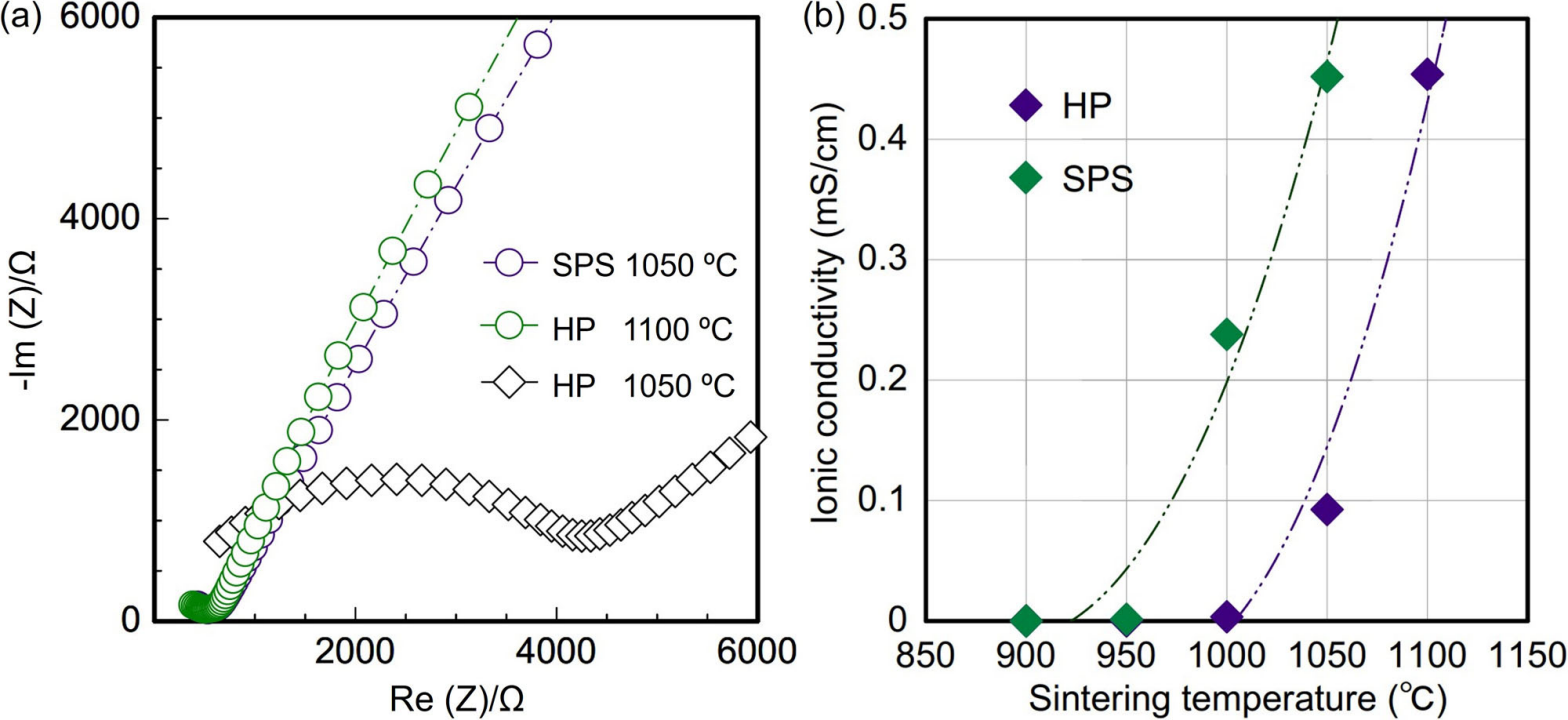
Ionic conductivity. a) EIS Nyquist plots of sintered Al‐LLZO pellets, b) Ionic conductivity as a function of sintering temperature.

EIS measurement was conducted to evaluate the RT ionic conductivity of Al‐LLZO pellets sintered via HP and SPS. As shown in Figure 5(a), the impedance spectra exhibit characteristic semicircles and tails, indicative of ionic conduction. The calculated ionic conductivity values, summarized in **Table** **3** and Figure 5b, reveal a clear positive correlation between sintering temperature and ionic conductivity.

**Table 3:** RT ionic conductivity of Al‐LLZO pellets sintered by HP and SPS.

Sintering temperature [°C]	950	1000	1050	1100
Ionic conductivity [mS cm^−1^] (HP)	0.000065	0.0035	0.093	0.45
Ionic conductivity [mS cm^−1^] (SPS)	0.0015	0.24	0.45	

For HP‐processed pellets, ionic conductivity increased with increasing sintering temperature from 900 to 1100 °C, which is attributed to improved densification. Similarly, SPS‐processed pellets exhibited an increase in ionic conductivity with increasing sintering temperature from 900 to 1050 °C. However, at higher temperatures, SPS‐processed pellets became prone to thermal cracking, probably because the rapid heating rate is more likely to generate larger residual stresses that lead to cracking. The enhanced conductivity observed up to 1050 °C is similarly attributed to improved densification. Overall, both HP and SPS enable the preparation of Al‐LLZO pellets with high ionic conductivity (>0.4 mS cm^−1^) within a short sintering duration of 5 min. While higher sintering temperatures generally improve conductivity by promoting densification, precise temperature control is essential to mitigate Li loss, prevent excessive grain growth, minimize phase decomposition, and optimize the ionic transport properties of Al‐LLZO. It should be noted that up to 1000 °C, the ionic conductivity of HP‐sintered pellets only reached 0.004 mS cm^−1^, while that of SPS‐sintered pellets already reached 0.2 mS cm^−1^. This is very likely attributed to a more uniform and elevated temperature distribution within the graphite die during SPS sintering, leading to enhanced densification and reduced grain boundary resistance. The ionic conductivity of HP‐sintered pellets drastically increased to ≈0.45 mS cm^−1^ at 1100 °C. Thus, SPS is more efficient in densification than HP at lower temperatures. Nevertheless, at optimal sintering conditions, LLZO pellets sintered via SPS only exhibited slightly higher relative density and ionic conductivity compared to those sintered via HP at the same nominal pressure and temperatures (Figures 3, 4, 5; Figures S3–S8, Supporting Information). This discrepancy suggests that despite the same measured or nominal sintering temperature at the outer circumference of the graphite die, the actual sintering temperature experienced by the LLZO inside differs between the two methods, with a high possibility that SPS generates a higher internal temperature in graphite dies compared to HP. Overall, the RT ionic conductivities of the HP‐ and SPS‐sintered Al‐doped LLZO under optimal conditions in this study are comparable to the reported values in the literature (0.1–1 mS cm^−1^).^[^ ^1^, ^39^, ^40^ ^]^

**Figure 6:**
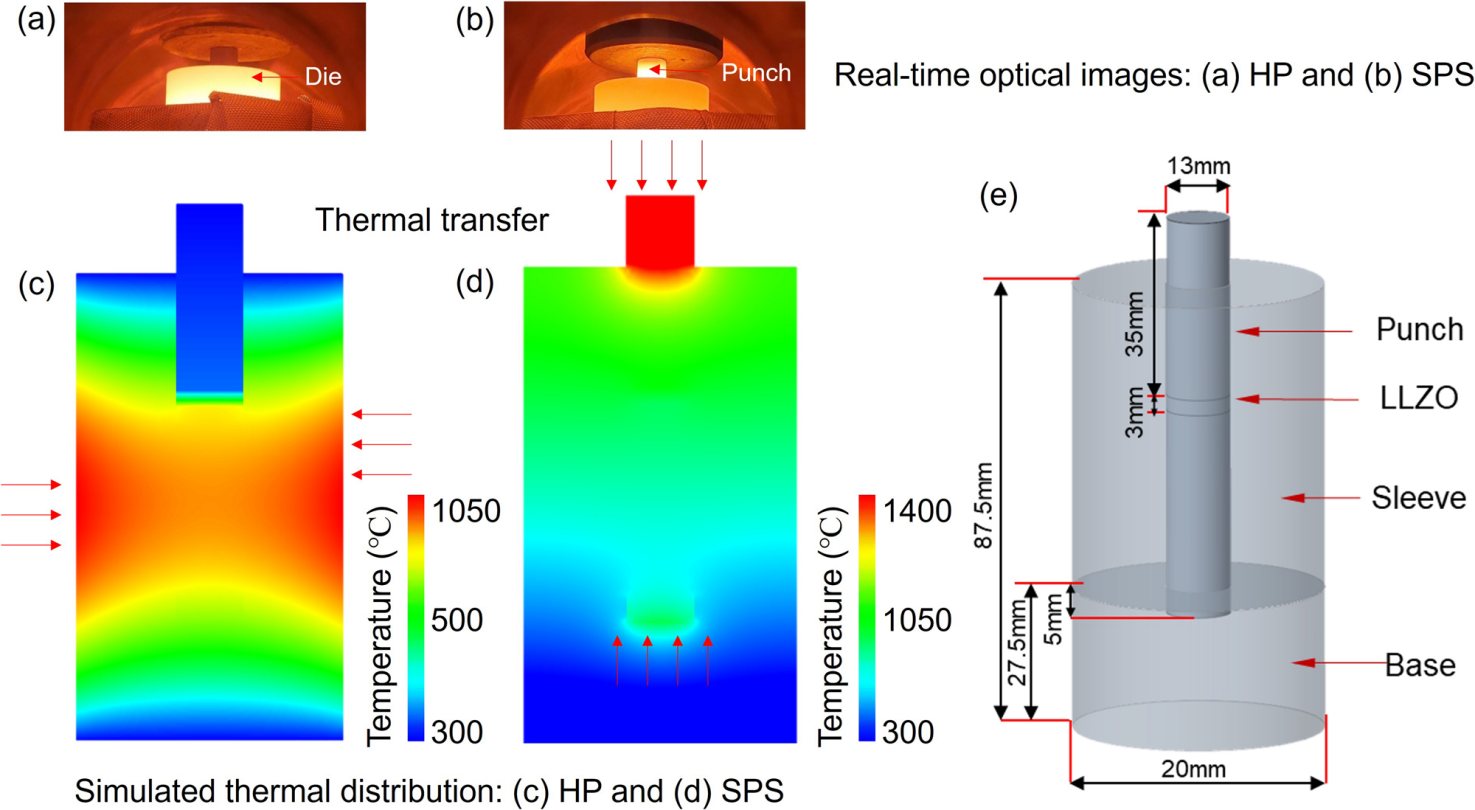
Thermal distribution. a) and b) Real‐time optical images of graphite dies during HP and SPS at 1050 °C, respectively, c) and d) Simulated thermal distribution during sintering after 50 seconds (the time required for the inner part of the graphite die to reach 1050 °C), e) Schematic representation of the dimensions and relative positions of the graphite dies and the cold‐pressed LLZO green body during sintering.

**Figure** **6**a,b shows real‐time optical images of the graphite dies during HP and SPS at 1050 °C, respectively. The graphite die in HP appears significantly brighter, indicating a higher temperature compared to the graphite punch. In contrast, the graphite punch in SPS (Figure 6b) shows a much brighter appearance than the graphite die, suggesting that the punch temperature is higher than that of the die. These observations suggest a distinct difference in thermal transfer direction between HP and SPS. During HP, the highest temperature is observed at the outer circumference of the graphite die, proximal to the heating coil surrounding the graphite die. Thus, heat transfer predominantly occurs from the die's outer surface toward the interior, where the cold‐pressed LLZO green body is situated. On the other hand, in SPS, the thermal transfer primarily occurs from the upper part of the graphite punch towards the graphite die.

To further understand the thermal behavior during sintering, heat conduction within the graphite punches and dies was simulated using the finite element method (FEM), as implemented in Abaqus (version 2016). The graphite punches, dies, and LLZO samples were discretized using eight‐node linear heat transfer elements (DC3D8) with a mesh size of 0.5 mm, resulting in ≈380 000 finite elements in total. A general contact interaction was defined between all components to model thermal interactions at the interfaces. The heat source was applied as a specified heat flux based on experimental observations. For HP, the heat source was defined as the outer part of the graphite die, reflecting its proximity to the heating coil. In SPS, the heat sources were defined as the graphite punches, consistent with the observed temperature distribution. The governing equation for thermal transfer during the sintering process can be described as follows:^[^ ^41^ ^]^

(5) $$\rho C_{P}\frac{\partial T}{\partial t}-\nabla q=\rho C_{P}\frac{\partial T}{\partial t}-\nabla \left(-k\nabla T\right)=0$$

where ρ is the material density, *C_P_* is the heat capacity, T is the temperature, t is the time, q is the thermal flux, and k is the thermal conductivity. The simulated thermal distribution results, as shown in Figure 6c,d reveal that the thermal transfer patterns differ significantly between HP and SPS. In HP, the temperature of the pelletized LLZO is lower (≈200 °C) than the nominal sintering temperature, measured at the outer circumference of the die. In contrast, in SPS, the temperature applied to the pelletized LLZO is nearly equal to the measured circumference temperature, indicating a more uniform thermal distribution, as shown in Figure 6d. The simulated temperature distributions within the dies, as shown in Figure 6c,d are consistent with the actual thermal behavior and closely match the temperatures measured during experiments. The thermal simulation parameters for the graphite mold and LLZO are summarized in **Table** **4**.

**Table 4:** Parameters of graphite mold and LLZO for thermal simulation.^[^ ^28^, ^41^, ^42^, ^43^ ^]^

Material/Constants	LLZO	Graphite (EX‐60)
Density, ρ [g cm^−3^]	5.11	1.8
Young's modulus, E [MPa]	154.9	12.1
Poisson's ratio, ν	0.24	0.2
Specific heat capacity, *C_P_* [J/kg/K]	0.81 × 10^−6^	711
Heat conductivity, *k_T_* [W/m/K]	1.5	110
Heat expansion coefficient, α [K^−1^]	3.27 × 10^−6^	5 × 10^−6^

Although SPS is associated with potential thermal inhomogeneity due to localized Joule heating, particularly in large or asymmetrically configured systems,^[^ ^22^ ^]^ the simulation and experimental results in this study indicate that, under specific conditions, SPS achieves a more uniform thermal distribution than induction‐based HP, which is typically considered fast and thermally uniform.^[^ ^26^ ^]^ The enhanced homogeneity, combined with a slightly higher effective sintering temperature, contributes to the improved densification and RT ionic conductivity observed in the SPS‐sintered LLZO. The improved thermal behavior is likely attributed to the die geometry and sample positioning. As shown in Figure 6e, the LLZO green body is centrally positioned within a compact and symmetric graphite punch‐die assembly, which facilitates efficient axial heat transfer and minimizes radial gradients, enabling a rapid and uniform temperature rise. In contrast, HP relies on external die wall heating, which can introduce thermal lag and larger radial inhomogeneity, as evidenced in Figure 6c. The thermal distribution in Figure 6d confirms a highly uniform temperature distribution in the upper half of the graphite punch and die region. These findings underscore that thermal uniformity in both SPS and HP is not solely determined by the sintering technique itself but is strongly dependent on tooling design, sample geometry, and placement.

A direct comparison of the maximum density and ionic conductivity values achieved by HP and SPS reveals only marginal differences, suggesting that the fundamental sintering mechanisms remain similar. Furthermore, the optimal sintering temperature, at which the highest ionic conductivity is achieved, is only 50 °C lower for SPS than for HP, indicating that the role of pulsed current heating in sintering LLZO is relatively insignificant.

**Figure 7:**
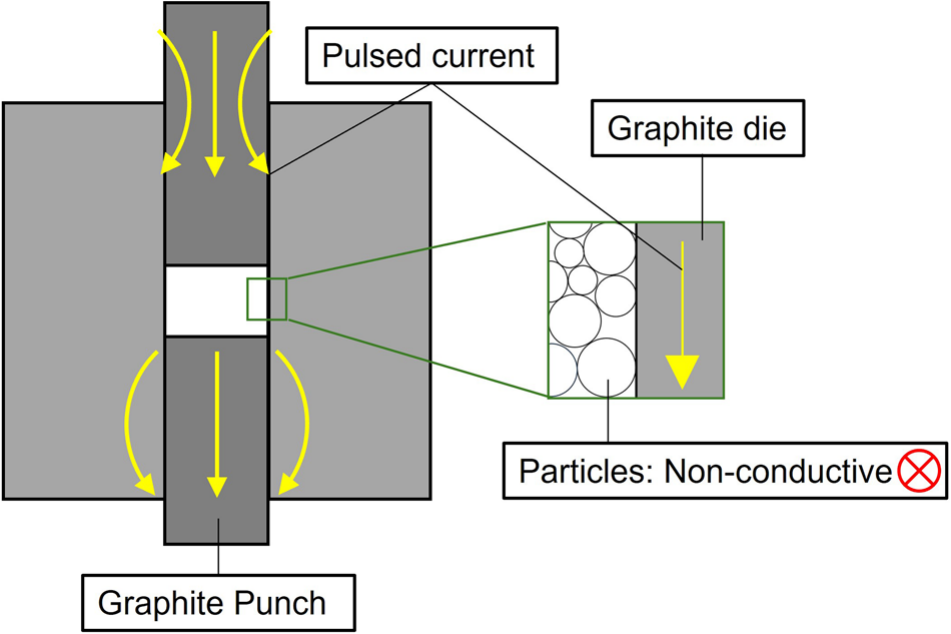
Schematic diagram showing pulsed current pathways during SPS of electrically nonconductive materials, such as LLZO.

Previous studies have investigated the influence of pulsed current on SPS sintering.^[^ ^22^, ^44^ ^]^ Their findings indicate that when sintering electrically nonconductive materials, the pulsed current predominantly flows through the graphite die rather than the powder compact itself. A similar phenomenon is expected in LLZO sintering, as illustrated in **Figure** **7**, where the pulsed current bypasses the LLZO powder compact, resulting in rapid heating of the die rather than direct effects on the LLZO particles. For conductive materials, SPS can induce localized spark plasma effects, such as surface cleaning of particles and enhanced diffusion, which accelerate sintering.^[^ ^22^ ^]^ However, since LLZO is an electrically nonconductive material, such effects are unlikely to occur. Consequently, sintering in SPS is primarily driven by thermal energy and mechanical pressure, similar to HP. The minor difference in optimal sintering temperatures, estimated to be 50–200 °C, between SPS and HP in this study further supports this conclusion, indicating that pulsed current heating does not fundamentally alter the sintering mechanism of LLZO but primarily affects the temperature distribution within the graphite die.

Besides thermal homogeneity, SPS can also influence material transport behavior through electric‐field‐induced phenomena, especially in electronically conductive systems. For example, in electrically conductive thermoelectric Zn_4_Sb_3_, the high mobility of Zn^2+^ under an applied electrical field during SPS has been shown to induce directional cation migration, causing phase separation and compositional gradients.^[^ ^45^ ^]^ In contrast, direct electromigration of the highly mobile Li⁺ during SPS is minimal as LLZO is a Li‐ion conductor with negligible electronic conductivity. However, at elevated temperatures, thermally driven Li+ migration and volatilization can occur.^[^ ^46^ ^]^

## 4 Conclusion

This study demonstrates that both induction hot pressing (HP) and spark plasma sintering (SPS) can effectively densify Al‐doped LLZO, achieving a high relative density of ≈98% and a high RT ionic conductivity of 0.45 mS cm^−1^ within a short sintering duration of only 5 min. The optimal sintering temperatures for HP and SPS are 1100 and 1050 °C, respectively, indicating a relatively small difference. The optimal sintering pressure is uniformly 25 MPa for both HP and SPS. Under proper conditions, HP can essentially match SPS, and the small edge for SPS at lower temperatures is simply due to heating efficiency, but the fundamental sintering mechanism remains unchanged due to LLZO's intrinsically nonconductive nature, which limits direct effects from the pulsed current. The key difference between HP and SPS lies in the spatial temperature distribution, with SPS achieving a more uniform and higher effective sintering temperature distribution due to more localized die heating.

## Conflict of Interest

The authors declare no conflict of interest.

## Author Contributions

M.F., Y.L., E.J.C., and J.W. performed formal analysis. J.W. and E.J.C. contributed to visualization. K.H., M.F., E.J.C., and Y.L. carried out investigations. G.L., Y.S.S., H.K., and E.J.C. provided supervision. G.J.S. and E.J.C. contributed to conceptualization. G.J.S., H.K., and E.J.C. performed validation. Y.S.S. and H.K. provided resources. H.K. and E.J.C. managed project administration. E.J.C. acquired funding. M.F. and E.J.C. contributed to writing.

## Supporting information



Supporting Information

## Data Availability

The data that support the findings of this study are available from the corresponding author upon reasonable request.
