# Supplementary material for: High‐Temperature Sintering of Garnet Solid Electrolyte Li7La3Zr2O12: A Comparative Study of Induction Hot Pressing and Spark Plasma Sintering
Source: Small. 2025 Aug 28;21(41):e06257. doi: 10.1002/smll.202506257 (PMC12530038; doi:10.1002/smll.202506257)
Supplement: Supplementary file 1 — Supporting Information [file SMLL-21-e06257-s001.docx]

**Supporting Information**

This Supporting Information provides expanded datasets and characterization results that reinforce the conclusions of the main manuscript. In particular, additional analyses were conducted in direct response to reviewer comments, ensuring clarity and completeness.


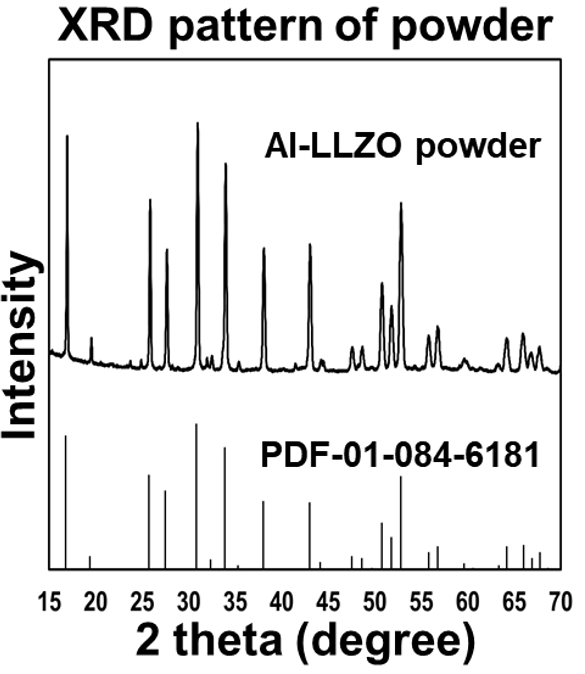
The supplementary dataset includes:

Fig. S1 The XRD pattern of as-received Al-doped LLZO powder

The XRD pattern of the as-received Al-doped LLZO powder in Fig.S1 confirms the formation of a typical cubic LLZO (c-LLZO) phase, indicating high phase purity prior to sintering. In addition to the main reflections of LLZO, a minor peak at approximately 24° was detected, which can be attributed to residual Li₂CO₃. Such carbonate impurities are commonly introduced during LLZO synthesis when Li₂CO₃ is used as a precursor, and their presence on LLZO surfaces is well documented as a result of exposure to atmospheric CO₂ and moisture.

Fig. S2 is the figure combined HP and SPS apparatus used in this study. This figure shows the hybrid sintering system capable of operating in both hot pressing (HP) and spark plasma sintering (SPS) modes. In both cases, pressure was applied through a load-controlled servo press integrated into the machine, ensuring stable and reproducible compaction during sintering. The heating pathways, however, differ between HP and SPS. In HP mode, heating is achieved by induction of the graphite die, whereas in SPS mode, pulsed current produces rapid Joule heating of the die and punches. These distinct thermal pathways lead to subtle but important differences: while both HP and SPS can reach nearly identical densification under optimal conditions, SPS tends to achieve a higher effective sintering temperature at the same nominal setpoint. At lower sintering temperatures, where both methods result in relatively porous microstructures, the more localized and efficient heating in SPS enhances particle bonding and thereby yields higher ionic conductivity compared with HP. This fundamental
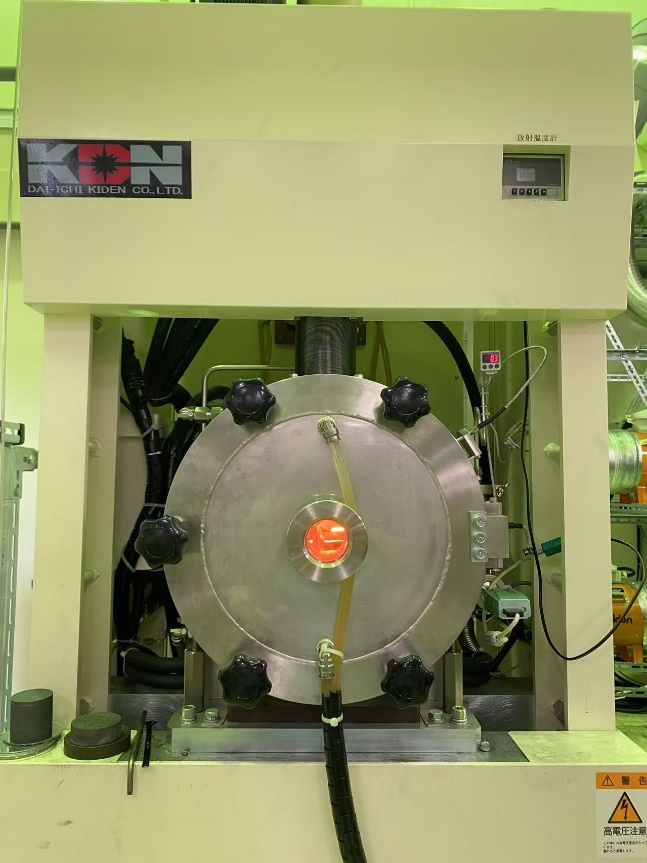
distinction underpins the performance comparison discussed in the main manuscript.

Fig. S2 The combined HP and SPS device for this study

Figs. S3–S8 present the microstructural evolution of Al-doped LLZO pellets processed by hot pressing (HP, 950–1150 °C; Figs. S3–S6) and spark plasma sintering (SPS, 950–1050 °C; Figs. S7–S8). The SEM images reveal clear densification and grain coarsening trends with increasing temperature. At lower temperatures (950–1000 °C), both HP- and SPS-sintered pellets exhibit significant porosity and irregular grain boundaries, reflecting incomplete densification. With further heating (1050–1150 °C), the microstructures become progressively denser, with larger and more uniformly bonded grains.

EDS elemental mappings confirm homogeneous distributions of La, Zr, and Al across the examined regions, validating that no significant elemental segregation occurred during sintering. However, as noted in the response to reviewer comments, the resolution of EDS mapping on fractured surfaces is somewhat limited due to the uneven topography. Nevertheless, the combination of SEM and EDS provides reliable evidence of the evolution of grain connectivity and pore elimination under different sintering conditions.

A comparative assessment shows that SPS promotes faster pore shrinkage and particle bonding at relatively lower nominal temperatures (e.g., 950–1000 °C) due to its distinct localized and rapid heating of the graphite mold. This explains why SPS-sintered pellets already exhibit higher relative densities and improved ionic conductivity in this regime. In contrast, HP requires higher nominal temperatures (≥ 1050 °C) to achieve comparable densification. At 1100–1150 °C, both techniques yield nearly fully dense microstructures (~98% relative density), highlighting that HP can essentially match SPS when optimized.

Together, these results substantiate the manuscript’s conclusion that both HP and SPS can rapidly densify LLZO within 5 minutes, while also clarifying the subtle differences in densification kinetics and microstructural evolution between the two methods.


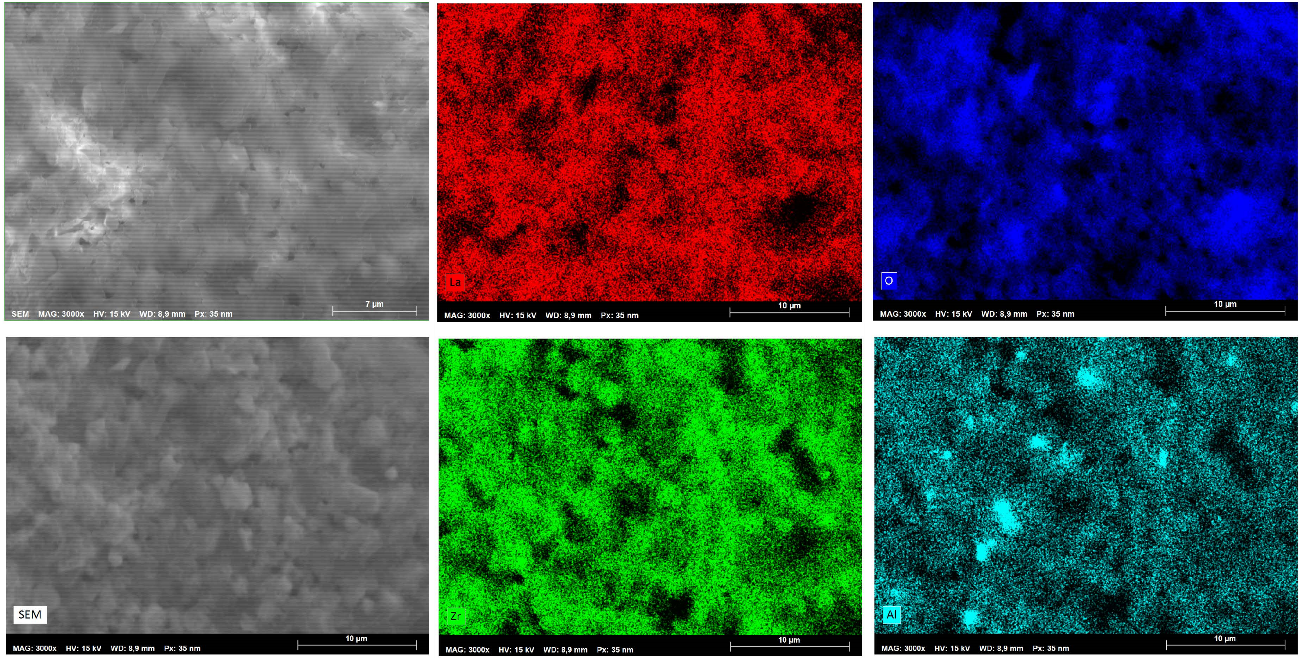


Fig. S3 SEM images and corresponding EDS mappings of Al-LLZO pellet HP sintered at 950 °C


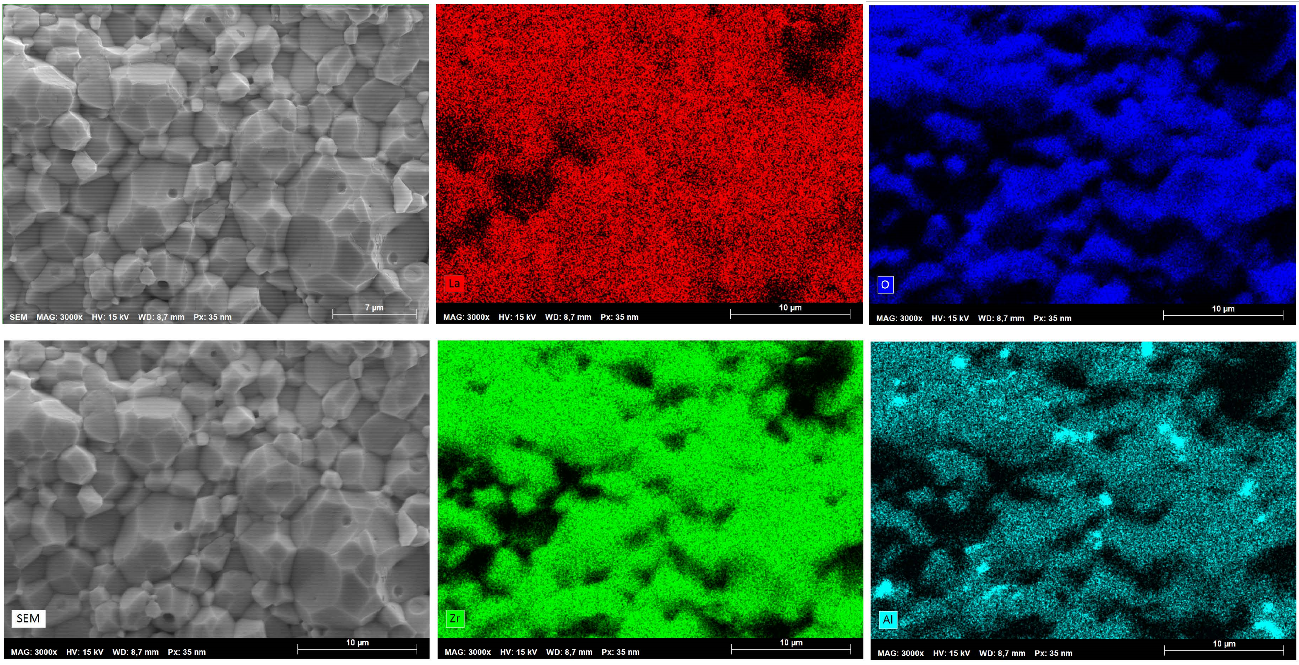


Fig. S4 SEM images and corresponding EDS mappings of Al-LLZO pellet HP sintered at 1050 °C


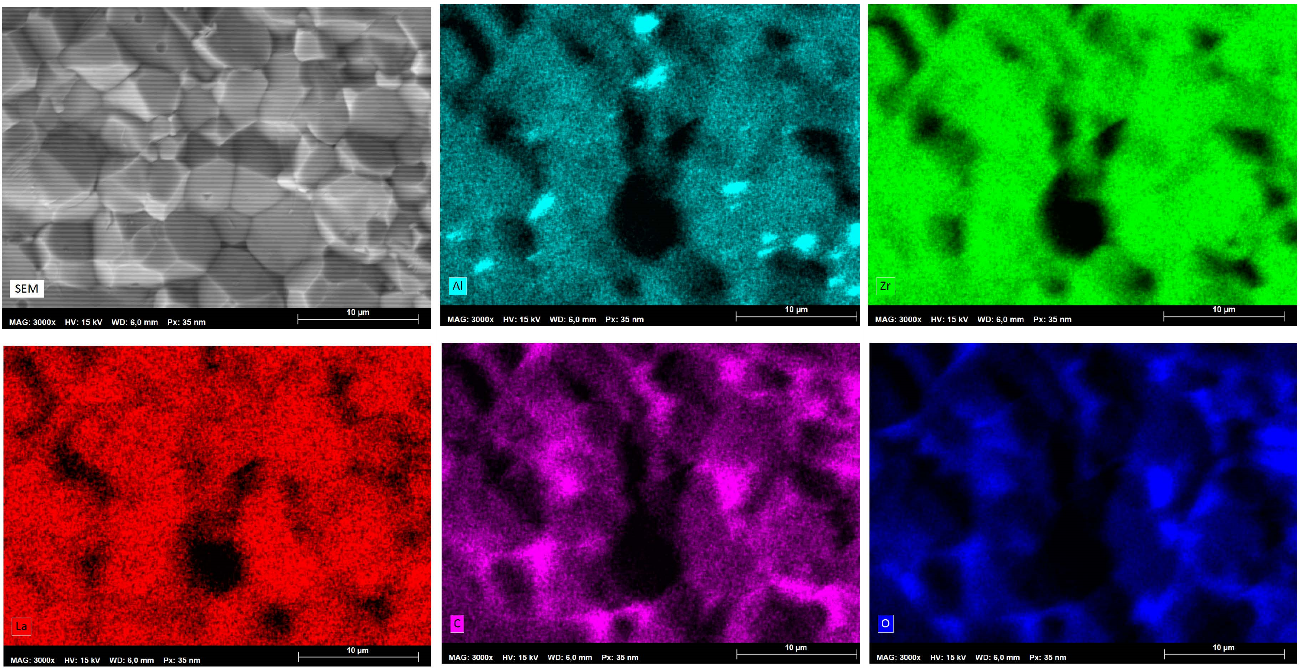


Fig. S5 SEM images and corresponding EDS mappings of Al-LLZO pellet HP sintered at 1100 °C


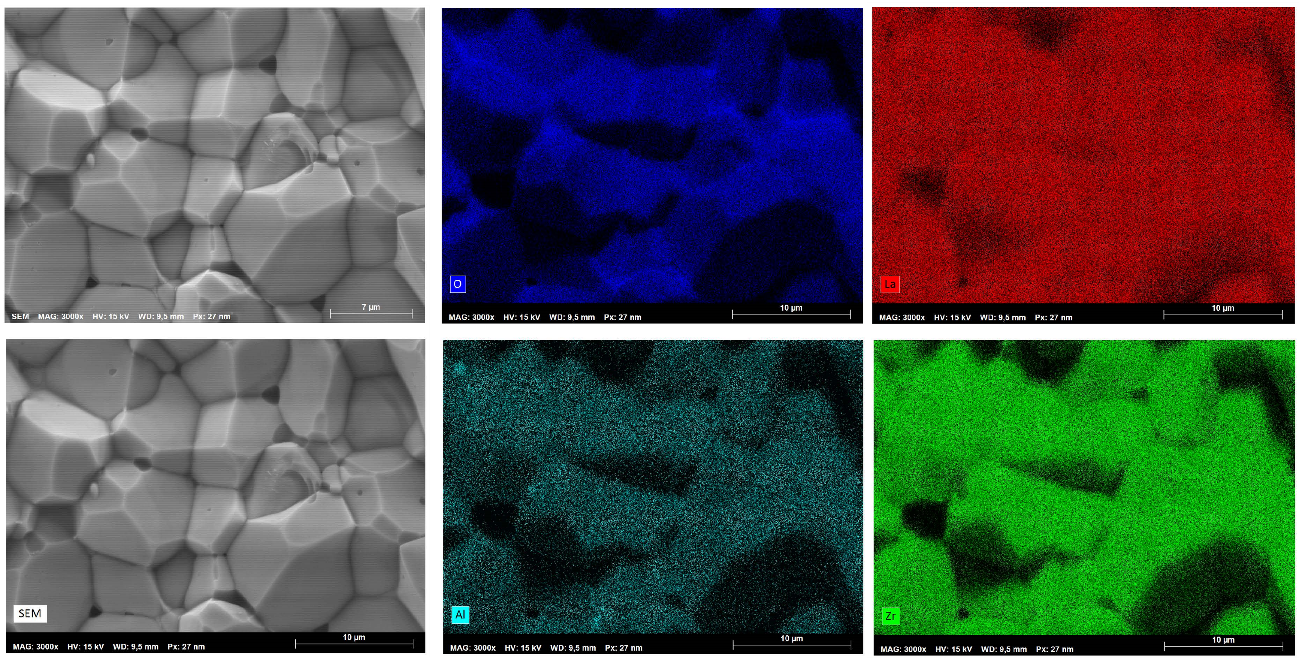


Fig. S6 SEM images and corresponding EDS mappings of Al-LLZO pellet HP sintered at 1150 °C


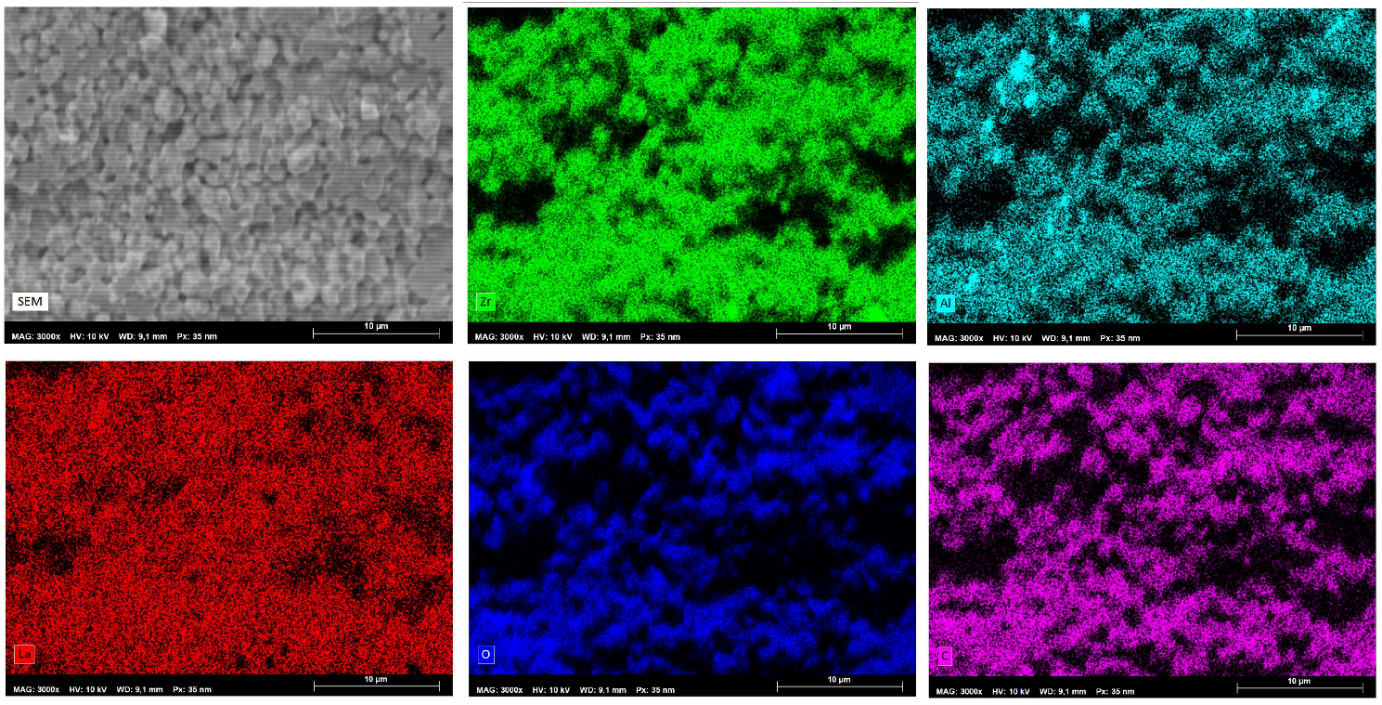


Fig. S7 SEM images and corresponding EDS mappings of Al-LLZO pellet SPS sintered at 950 °C


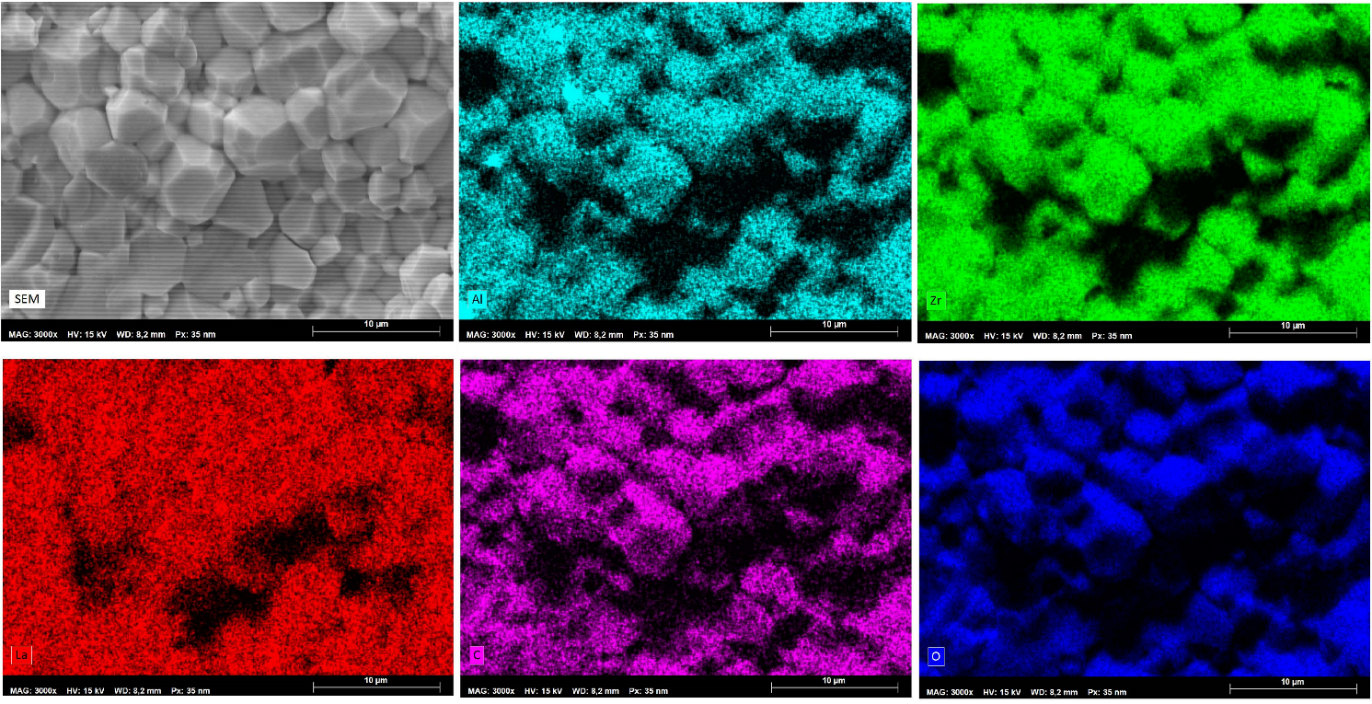


Fig. S8 SEM images and corresponding EDS mappings of Al-LLZO pellet SPS sintered at 1050 °C


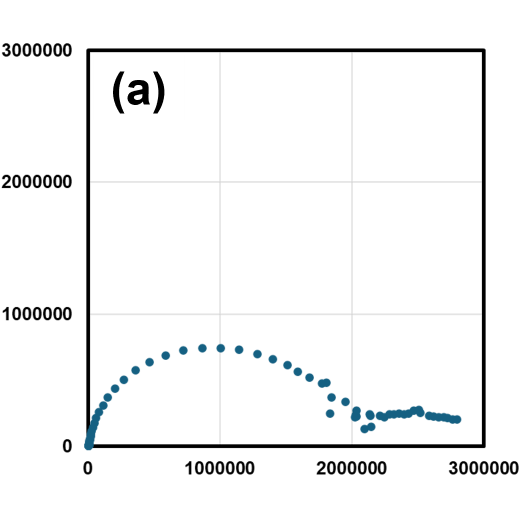

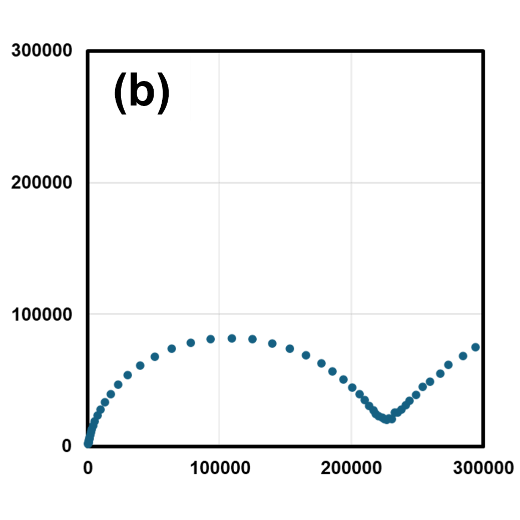

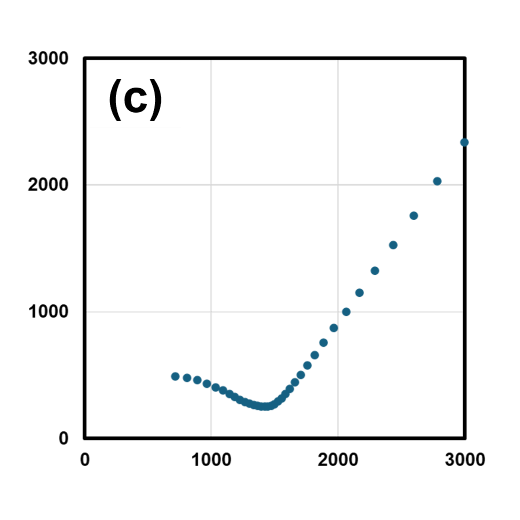

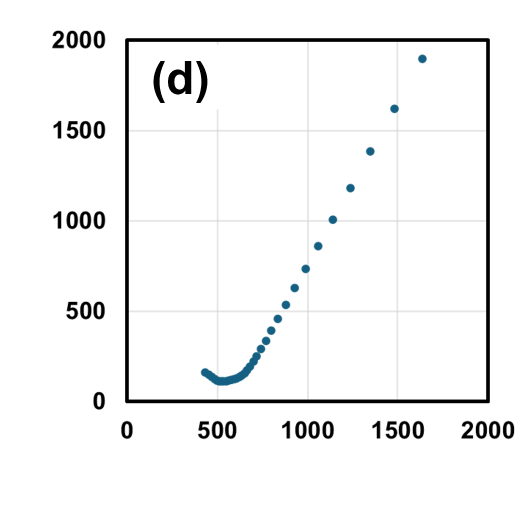


Fig. S9 Impedance plots of Al-LLZO pellets sintered by SPS (a)900 ℃, (b)950 ℃, (c)1000 ℃, (d)1050 ℃. The thicknesses of the pellets are as follows: SPS-900: 2.42mm, SPS-950: 2.58mm, SPS-1000: 2.66mm, SPS-1050: 2.56mm. The Au-coated area on both sides of the pellets is 0.7854 cm^2^.


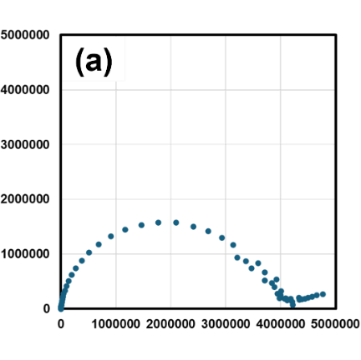

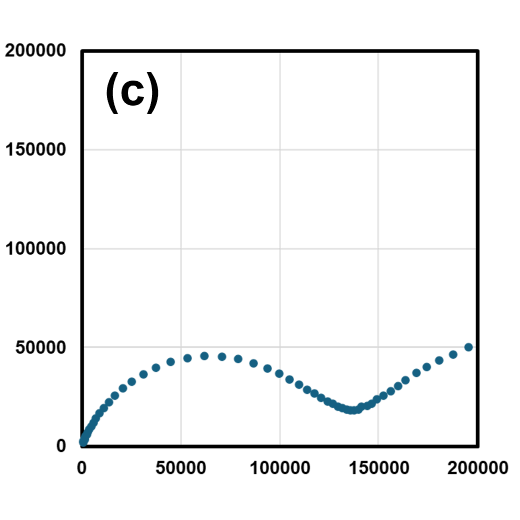

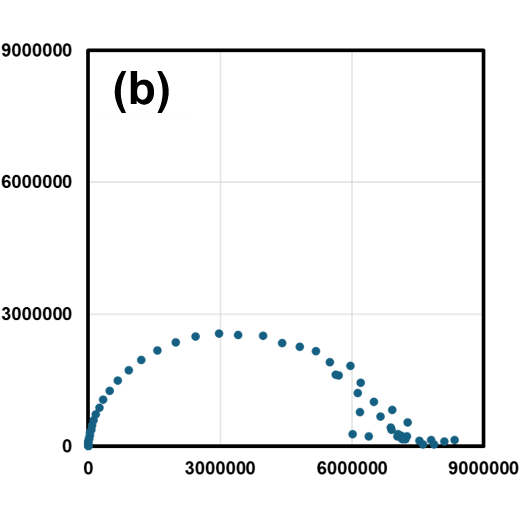

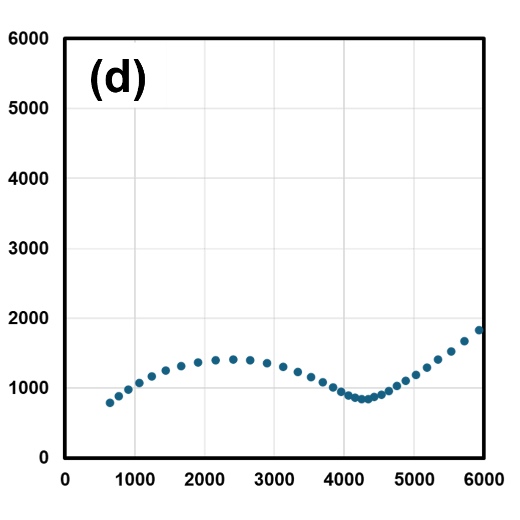

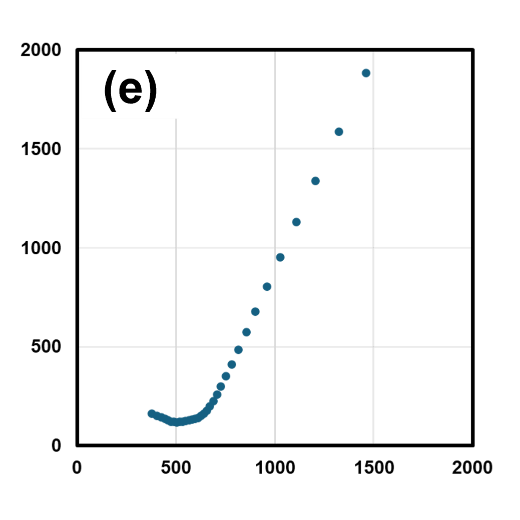

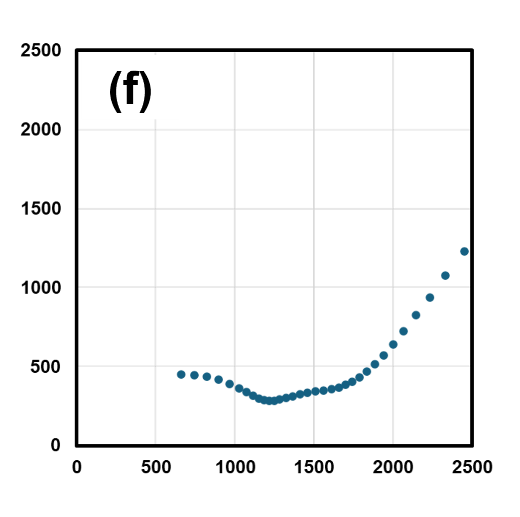


Fig. S10 Impedance plots of LLZO pellets made by **HP** (a)900 ℃, (b)950 ℃, (c)1000 ℃, (d)1050 ℃,(e)1100 ℃, (f)1150 ℃. The thicknesses of the pellets are as follows: HP-900: 2.59mm, HP-950: 3.44mm, HP-1000: 3.15mm, HP-1050: 2.91mm, HP-1100: 2.57mm, HP-1150: 2.76mm. The Au-coated area on both sides of the pellets is 0.7854 cm^2^.

These figures present the complete EIS datasets of Al-doped LLZO pellets processed via spark plasma sintering (SPS, Fig. S9) and hot pressing (HP, Fig. S10) at different sintering temperatures. For SPS, spectra are shown for pellets sintered at 900, 950, 1000, and 1050 °C. For HP, spectra are provided for pellets sintered across a wider temperature range (900–1150 °C). The corresponding pellet thicknesses and electrode areas are specified to ensure accurate conductivity calculations.

The Nyquist plots exhibit characteristic semicircular arcs at high to intermediate frequencies, followed by inclined tails at low frequencies, confirming dominant ionic conduction with blocking Au electrodes. With increasing sintering temperature, the semicircle diameter (bulk and grain boundary resistance) decreases significantly, reflecting improved densification, reduced porosity, and enhanced grain-to-grain connectivity. This trend is consistent with the SEM observations (Figs. S3–S8) and relative density measurements in the main text.

Together, the results presented here confirm that both induction hot pressing (HP) and spark plasma sintering (SPS) can densify Al-doped LLZO to nearly full density (~98%) within only 5 minutes, producing high ionic conductivity (>0.45 mS cm⁻¹). The data also emphasize subtle differences: SPS shows advantages at lower sintering temperatures due to localized, rapid heating, whereas HP offers a more stable processing route with reduced risk of thermal cracking.

The figures provided below (Figs. S1–S10) should be read in conjunction with the main text for a comprehensive understanding of the densification and electrochemical behavior of Al-doped LLZO.
